# Systematic Review of Efficacy and Safety of Avacopan in Real-World Clinical Practice

**DOI:** 10.1016/j.ekir.2025.103753

**Published:** 2025-12-31

**Authors:** Ilay Berke, Felix Keller, Clemens Untersulzner, Jae Il Shin, Peong Gang Park, Sarah Soyeon Oh, Jasper Callemeyn, Andreas Kronbichler

**Affiliations:** 1 Department of Internal Medicine IV, Nephrology and Hypertension, Medical University Innsbruck, Austria; 2 Department of Pediatrics, Yonsei University College of Medicine, Seoul, Republic of Korea; 3 The Center for Medical Education Training and Professional Development in Yonsei-Donggok Medical Education Institute, Seoul, Republic of Korea; 4 Severance Underwood Meta-research Center, Institute of Convergence Science, Yonsei University, Seoul, Republic of Korea; 5 Institute of Global Engagement and Empowerment, Yonsei University, Seoul, Republic of Korea; 6 Nephrology and Renal Transplantation Research Group, Department of Microbiology, Immunology and Transplantation, KU Leuven, Leuven, Belgium; 7 Department of Health, Medicine and Caring Sciences, Linköping University, Linköping, Sweden

**Keywords:** ANCA, avacopan, complement, treatment, vasculitis

## Abstract

**Introduction:**

Avacopan, a complement 5a receptor (C5aR) antagonist, is a therapeutic option for patients with antineutrophil cytoplasmic antibody (ANCA)-associated vasculitis (AAV), and is used as a steroid-sparing agent. The efficacy and safety of avacopan were established in the pivotal phase III ADVOCATE trial. However, there remains a paucity of real-world evidence to confirm these findings across diverse clinical settings and populations.

**Methods:**

We conducted a systematic review of 16 real-world studies evaluating the clinical outcomes of avacopan in patients with AAV. Using a meta-analytic approach, we compared efficacy and safety outcomes reported in these studies with those of the main trial. Key end points included clinical remission and incidence of adverse events.

**Results:**

The aggregated real-world data demonstrated that the time from diagnosis of AAV or relapse and initiation of avacopan was 24 days (range: 6–54 days). The clinical remission rates at 6 months as assessed in 215 patients were 89% (95% confidence interval [CI]: 0.84–0.93), whereas the rates of serious infection were 14% (95% CI: 0.10–0.18). We observed a heterogeneity between populations when hepatotoxicity was assessed in real-world cohorts, with this signal being particularly pronounced in Japanese populations.

**Conclusion:**

Avacopan has been found to demonstrate both safety and high efficacy in the treatment of AAV in real-world settings, with remission rates exceeding and serious infection rates comparable to those observed in clinical trial data. However, the higher incidence of hepatotoxicity in certain populations underscores the need for careful monitoring and pharmacovigilance studies to clarify risk factors and guide patient selection.

## Graphical abstract

**Figure:**
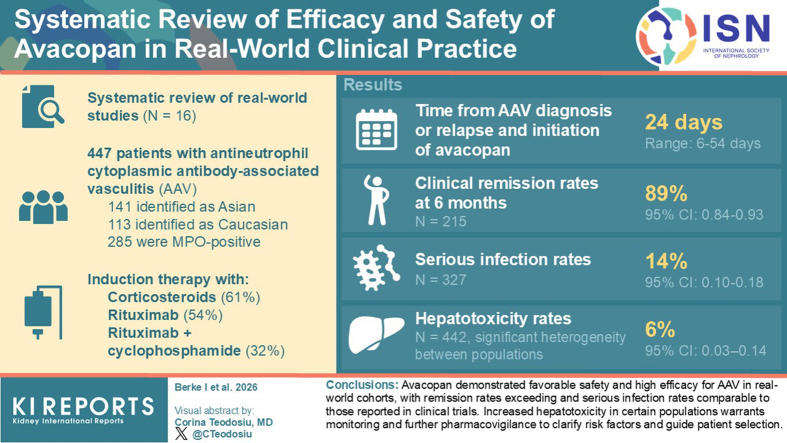

AAV is a rare, relapsing, and potentially life-threatening autoimmune disease marked by necrotizing inflammation of small- to medium-sized blood vessels, most commonly affecting the kidneys, lungs, and other vital organs, and comprising 3 main clinical phenotypes, namely granulomatosis with polyangiitis, microscopic polyangiitis, and eosinophilic granulomatosis with polyangiitis.^1^

AAV develops in genetically predisposed individuals, with environmental triggers initiating the process. ANCAs hyperactivate neutrophils, leading to the release of inflammatory mediators and an excessive formation of neutrophil extracellular traps.^2^ This overproduction damages vascular endothelial cells and stimulates further ANCA production, creating a self-perpetuating cycle central to disease development. This inflammatory response is further amplified by activation of the alternative complement pathway, especially through the C5a-C5aR axis, establishing a continuous proinflammatory loop that enhances both innate and adaptive immune responses.^3^

Current standard-of-care regimens for new-onset or relapsing AAV involve glucocorticoids (GCs) in combination with rituximab (RTX) or cyclophosphamide to induce remission, with RTX preferred in cases of relapsing granulomatosis with polyangiitis or microscopic polyangiitis.^1^^,^^4^^,^^5^ Although effective, these therapies are associated with substantial toxicity—particularly within the first year—including infections; metabolic disturbances; and long-term complications such as hypogammaglobulinemia, malignancy, and infertility.^6^ The high burden of treatment-related adverse events, especially in relapsing cases, has spurred interest in GC-sparing approaches.

Avacopan, an oral selective C5aR antagonist, blocks complement-driven neutrophil activation while preserving the membrane attack complex. In the phase 3 ADVOCATE trial,^7^ it matched high-dose GCs in inducing remission at 26 weeks, outperformed them in sustaining remission at 52 weeks, and significantly reduced steroid exposure, supporting its role as an effective GC-sparing therapy.

However, real-world data on avacopan remain limited, particularly in regions where AAV presents with different clinical profiles. This study aimed to evaluate the real-world efficacy and safety of avacopan in patients with AAV, with particular attention to treatment outcomes and adverse events across diverse patient populations.

## Methods

This study was conducted in accordance with the Preferred Reporting Project for Systematic Review and Meta-Analysis (PRISMA) guidelines and was registered at PROSPERO (https://www.crd.york.ac.uk/prospero/)(registration No: CRD420251032519).

### Search Strategy

We did a literature search in MEDLINE, Cochrane Library, and EMBASE on September 2025. The search strategy was not limited to any year of publication but to English. We used the string (("avacopan"[Supplementary Concept] OR "avacopan"[All Fields]) AND ("anti neutrophil cytoplasmic antibody associated vasculitis"[MeSH Terms] OR ("anti neutrophil"[All Fields] AND "cytoplasmic"[All Fields] AND "antibody associated"[All Fields] AND "vasculitis"[All Fields]) OR "anti neutrophil cytoplasmic antibody associated vasculitis"[All Fields] OR ("anca"[All Fields] AND "associated"[All Fields] AND "vasculitis"[All Fields]) OR "anca associated vasculitis"[All Fields])) AND ((fft[Filter]) AND (english[Filter])) for MEDLINE, and “avacopan AND (anca OR vasculitis)” for Cochrane Library, and (‘anca’/exp OR anca) AND (‘avacopan’/exp OR avacopan) for EMBASE.

### Study Selection

Two investigators (IB and AK) independently screened each citation. Every publication considered potentially relevant by either reviewer was retrieved for a full-text review.

The inclusion criteria were as follows: articles reporting patients aged > 18 years with either relapsing or newly diagnosed granulomatosis with polyangiitis, microscopic polyangiitis, proteinase 3–AAV, myeloperoxidase-AAV, or ANCA-negative AAV, a minimum of 5 patients included in the study, data representing real-world clinical outcomes, patients who have received ≥ 2 weeks of treatment with avacopan. Exclusion criteria were as follows: full-text article not available, article written in a language other than English.

**Figure 1:**
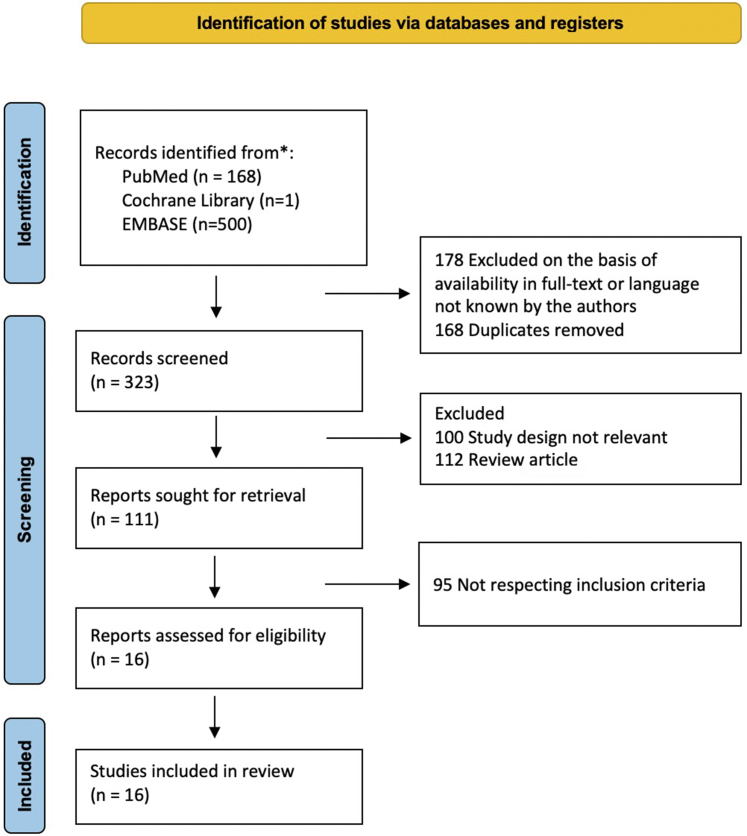
Preferred reporting items for systematic reviews and meta-analyses (PRISMA) flow diagram of the literature search.

We identified a total of 16 studies from the literature, published over a time span of 4 years. The PRISMA flow diagram is presented in Figure 1.

### Data Extraction

Extraction of data from eligible articles was performed using a standardized data collection form. The following data were extracted: age; sex and/or gender; race or ethnicity; ANCA type; new diagnosis or relapsing disease; type of induction therapy; time and duration of avacopan initiation; cumulative steroid dose; median steroid dose at avacopan initiation; clinical remission at 3, 6, and 12 months; serious adverse events or toxicities; need and reason for avacopan discontinuation; and mortality. Clinical remission was accepted as BVAS score of 0.

### Quality Assessment

The quality of the studies was assessed using the National Heart, Lung, and Blood Institute's tools for observational cohort and cross-sectional studies, and for case series studies. Nine studies were rated as good quality, and 7 as fair. However, the absence of a comparator implies an inherently high risk of bias^8^ (Supplementary Table S1A–C).

### Statistical Analysis

To provide a descriptive synthesis of the quantitative data across included studies and to acknowledge present heterogeneity, we calculated cluster-level and overall summary proportions. Event counts and denominators from each study were synthesized using a logit transformation with inverse-variance weighting within a random-effects framework. Studies that reported an outcome with 0 events were included in the pooling; the logit transformation accommodates 0 and 100% proportions without requiring continuity corrections. Studies that did not report a specific outcome (i.e., no data provided for that end point) were excluded from pooling for that end point only. To account for potential regional differences in clinical practice and population characteristics, studies were grouped by geographic region or country, which was modeled as a clustering variable with a shared random intercept.

As a sensitivity analysis, we fitted binomial generalized linear mixed models with a logit link, which similarly accommodates 0 and 100% proportions. The generalized linear mixed model results, which were concordant with the primary proportional meta-analysis, are presented in Supplementary Table S3.

These pooled proportions and CIs are intended as illustrative summaries rather than confirmatory effect estimates. Accordingly, we report heterogeneity statistics (τ^2^, and the *P*-value of Cochrane’s Q) solely to characterize between-study variability. No formal hypothesis testing or inferential conclusions were drawn.

Forest plots are presented to visually display the distribution, consistency, and magnitude of proportions across studies and regions. These descriptive aggregates are intended to support interpretation of clinical trial data by contextualizing real-world evidence from diverse observational settings.

All analyses were carried out using R version 4.5.0 (2025-04-11),^9^ with the meta package for proportional meta-analysis and the metafor package for generalized linear mixed model sensitivity analyses.

## Results

A total of 16 studies evaluating the safety and efficacy of avacopan in real-world settings were included, encompassing 447 patients overall. The majority of these studies were retrospective cohort studies (12/16, 75%) and multicenter in design (12/16, 77%). Five studies were conducted in Japan, whereas the remainder originated from European countries and the United States. Due to the inclusion of both single-center and multicenter studies, sample sizes varied considerably, ranging from 5 to 92 participants. The mean age of participants across studies ranged from 46 to 78 years. Key study characteristics are summarized in Table 1.^10^, ^11^, ^12^, ^13^, ^14^, ^15^, ^16^, ^17^, ^18^, ^19^, ^20^, ^21^, ^22^, ^23^, ^24^, ^25^

**Table 1:** Main characteristics of the included studies

Author	Publication year	Study design	Single/Multicenter	Study region	Number of patients	Mean age, yr	Male (%)
Tagami et al.^10^	2025	Retrospective cohort	Single center	Japan	21	NR	52.4
Mori et al.^11^	2024	Retrospective cohort	Multicenter	Japan	22	68	36.4
Draibe et al.^12^	2025	Retrospective cohort	Multicenter	Spain	29	NR	41.4
Zimmermann et al.^13^	2024	Retrospective cohort	Multicenter	Germany	39	64	54
Uchida et al.^14^	2024	Retrospective cohort	Multicenter	Japan	36	NR	38.9
Falde et al.^15^	2024	Retrospective cohort	Multicenter	United States	15	NR	40
Gabilan et al.^16^	2025	Retrospective cohort	Multicenter	France	31	NR	68
Zonozi et al.^17^	2024	Retrospective cohort	Multicenter	United States	92	59	36
Chalkia et al.^18^	2024	Case series	Multicenter	UK	8	64	37.5
van Leeuwen et al.^19^	2023	Prospective cohort	Multicenter	Australia, Germany, France, Ireland, the Netherlands, Switzerland, UK	30	NR	70
Gabilan et al.^20^	2022	Retrospective cohort	Multicenter	France	9	75	
van Leeuwen et al.^21^	2022	Case series	Single center	Netherlands	8	46	62.5
Kubota et al.^22^	2024	Case series	Single center	Japan	5	78	60
Takeuchi et al.^23^	2025	Retrospective cohort	Single center	Japan	57	NR	29.8
Eisinger et al.^24^	2025	Retrospective cohort	Multicenter	United States	15	NR	47
Assmann et al.^25^	2025	Retrospective cohort	Multicenter	Germany	30	59	50

NR, not reported.

Data on ethnicity were available in a limited number of studies. Of those reported, 141 patients were of Asian descent, and 113 were identified as Caucasian. The majority of patients were myeloperoxidase-positive in all studies, 285 patients in total (68%). Newly diagnosed patients constituted 64% of the analysis (215 patients). As part of induction therapy, 179 patients (61%) received pulse corticosteroids. RTX was used in 182 patients (54%), cyclophosphamide in 17 patients (5%), and a combination of RTX and cyclophosphamide in 107 patients (32%) (Supplementary Table S2).

Cumulative steroid dose data were available in 6 studies, ranging from 700 to 3090 mg. The median time from diagnosis or relapse to avacopan initiation was 24 (range: 6–54) days, whereas the daily corticosteroid dose at avacopan initiation varied between 8 and 50 mg across studies. Eight studies reported the duration of avacopan treatment, with median durations ranging from 39 days to 99 weeks. Complete steroid withdrawal rates varied widely, from 0% to 100%, with an average of 59% (Supplementary Figure S1).

**Figure 2:**
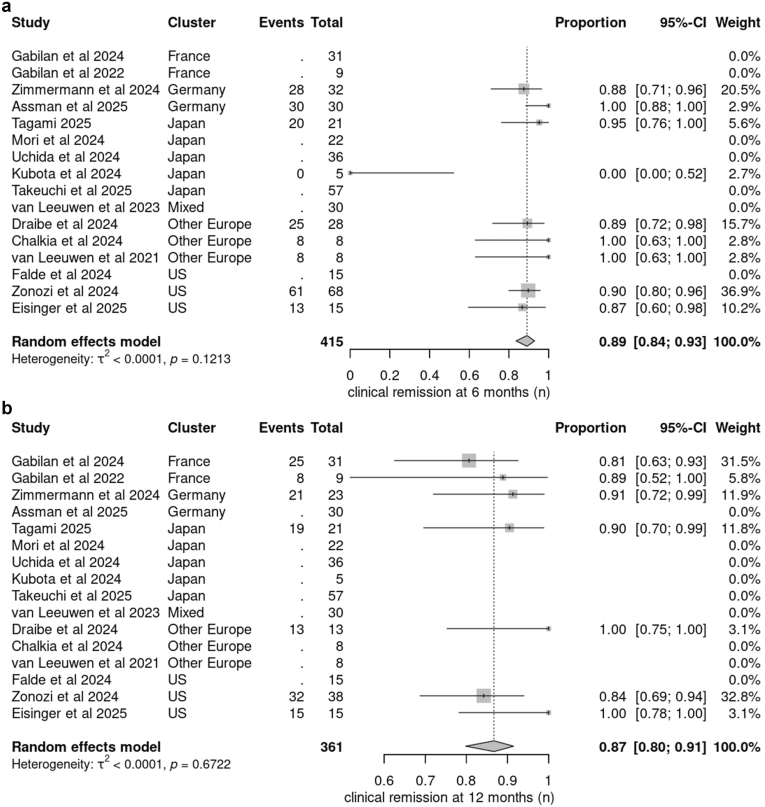

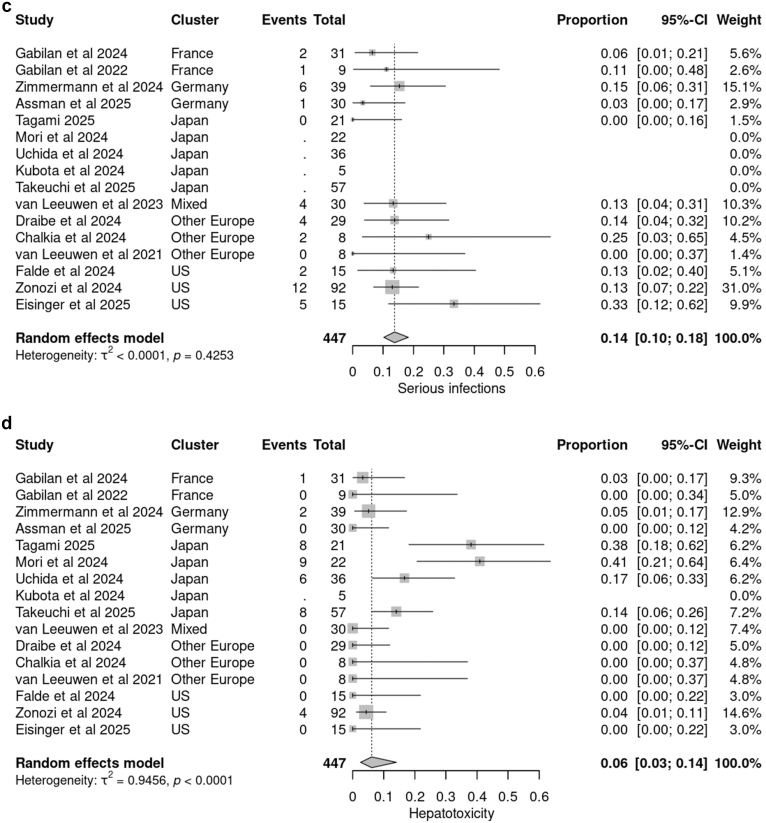
Forest plots were used to perform the pooled analysis. For binary variables, proportions were calculated, with statistical significance determined at a 95% CI. Each study’s weight is indicated by the size of the square next to its name, whereas the diamond represents the overall pooled result. Separate forest plots were generated for different outcomes, including (a) clinical remission at 6 months, (b) clinical remission at 12 months

Remission rates at 6 months were reported in 9 studies, and 12-month remission rates were reported in 7 studies. Notably, in 1 Japanese case series,^22^ none of the patients achieved remission at 6 months, contributing to substantial heterogeneity. Clinical remission rates at 12 months varied significantly among studies. The pooled proportion of patients achieving clinical remission at 6 months was 0.89 (95% CI: 0.84–0.93), based on 215 patients across included studies. At 12 months, the pooled remission proportion was 0.87 (95% CI: 0.80–0.91), reflecting consistent outcomes over time in real-world settings (Figure 2a and b). Relapse occurred in 18 patients (7%) without any significant difference among studies.

The pooled rate of serious infections was 0.14 (95% CI: 0.10–0.18), with pneumonia and urinary tract infections being the most frequently reported. No data were available regarding opportunistic pathogens. The proportion of hepatotoxicity events was 0.06 (95% CI: 0.03–0.14), though substantial heterogeneity was observed in the latter (Figure 2c). Hepatotoxicity was predominantly observed in the Japanese cluster, with a substantially higher incidence than in other clusters (Figure 2d). Additional serious adverse events potentially associated with avacopan included leukopenia or neutropenia (*n* = 10), gastrointestinal side effects (*n* = 8), age-related macular degeneration (*n* = 3), and rash (*n* = 2).

Avacopan was discontinued in 23% of patients (105/447), most commonly due to adverse events. Additional reasons for discontinuation included insurance-related issues, high cost, and lack of therapeutic response.

## Discussion

Avacopan, an oral selective C5aR antagonist, inhibits complement-mediated neutrophil activation without compromising the formation of the membrane attack complex. In the phase 3 ADVOCATE trial, avacopan demonstrated noninferiority to high-dose GCs for induction of remission at week 26 and superiority in sustaining remission at week 52, alongside a significantly reduced cumulative GC burden.^7^ These findings support the utility of avacopan as an effective GC-sparing agent, particularly in patients with a high risk of steroid-related toxicity.

In our analysis, the pooled remission rates with avacopan therapy were notably higher than those reported in the pivotal ADVOCATE trial, with 89% of patients achieving remission at 6 months and 86% at 12 months, compared with 72.3% and 65.7%, respectively, in the original trial population. This higher remission rate may be attributable to the continued use of maintenance immunosuppressive therapy following induction and to higher cumulative exposure to GCs relative to the ADVOCATE protocol. In contrast, a case series of 5 patients with longstanding AAV (disease duration: 1–5 years) reported no clinical remission with avacopan.^22^ The drug was started in 4 patients because of relapse and in 1 because of GC intolerance, with treatment durations ranging from 6 to 52 weeks. The authors suggested that combining avacopan with B-cell–depleting agents such as RTX may be necessary to achieve better clinical outcomes in such cases. Indeed, a subanalysis of ADVOCATE focusing on the 107 patients who received RTX alongside avacopan reported remission rates of 77.6% and 71.0%, in part supporting this assumption.^26^

In the study by Draibe *et al.*,^12^ a high remission rate was observed at 6 months. Although follow-up durations varied among patients, all 13 individuals with 12-month data had achieved a BVAS of 0. Notably, this study included a comparison with a historical control group—unlike other reports—and demonstrated significantly lower cumulative GC exposure in the avacopan-treated cohort. However, no statistically significant differences were found in relapse rates or estimated glomerular filtration rate (eGFR) at 12 months. The lack of difference in renal outcomes was attributed to the small sample size and lower baseline proteinuria in the control group.

Although rapidly progressive glomerulonephritis is a key feature of AAV, the ADVOCATE trial excluded patients with severe renal impairment (eGFR < 15 ml/min per 1.73 m^2^). However, a *post hoc* analysis of 50 patients with baseline eGFR ≤ 20 ml/min per 1.73 m^2^ (27 patients receiving avacopan, 23 prednisone) revealed significantly greater improvement in the avacopan group at week 52 (mean increase: 16.1 vs. 7.7 ml/min per 1.73 m^2^), along with a more favorable safety profile.^27^ Similarly, Zonozi *et al.*^17^ evaluated 21 patients with eGFR < 15 ml/min per 1.73 m^2^ and found greater improvements in renal function at weeks 26 and 52 than in those with higher baseline eGFR (mean increases of 19.4 and 25.1 ml/min per 1.73 m^2^, respectively). Safety outcomes were comparable across groups. Among 9 dialysis-dependent patients, 5 achieved clinically meaningful renal recovery. Zimmermann *et al.*^13^ likewise reported a marked eGFR increase in patients with baseline < 15 ml/min per 1.73 m^2^ (from 8 to 35 ml/min per 1.73 m^2^). Additional case series have demonstrated favorable renal outcomes with avacopan, either as monotherapy or in combination, in patients with advanced kidney dysfunction.^28^ Considering different renal end points, a subgroup analysis of 268 patients with renal involvement in the ADVOCATE trial demonstrated faster reduction of proteinuria and hematuria in the avacopan group than in the prednisone taper group.^29^

In our analysis, a notable finding was the markedly higher rate of hepatotoxicity in the Japanese cohort than in studies conducted in Europe and North America. In the ADVOCATE trial, 9 patients in the avacopan group developed abnormal liver function tests, one of whom was from the Japanese subpopulation.^30^ In addition, a recent pooled safety analysis combining data from 3 avacopan clinical trials reported serious hepatic adverse events in 4.4% of avacopan-treated patients versus 2.8% in the control group.^31^ In 2 of these trials, the participant population was predominantly White, whereas 10.2% of patients in the avacopan arm of the ADVOCATE trial were of Asian descent. Among patients who experienced hepatic adverse events, avacopan was either temporarily interrupted or permanently discontinued in 7 out of 10 cases. All liver-related events resolved without lasting complications. In our real-world dataset, avacopan was discontinued in 34 out of 38 patients who developed hepatotoxicity, with 1 death because of vanishing bile duct syndrome. Several other cases of vanishing bile duct syndrome have been described from Japan following avacopan-containing therapy for AAV.^32^^,^^33^ Although concomitant trimethoprim-sulfamethoxazole use may have contributed to drug-induced liver injury (DILI), resolution after avacopan discontinuation suggests it was likely the primary cause.^10^^,^^11^^,^^13^^,^^14^ Both Tagami *et al.*^10^ and Mori *et al.*^11^ reported older age as significantly associated with DILI; the latter also showed that patients with DILI had a higher prevalence of myeloperoxidase-ANCA positivity, and those with total bilirubin elevation had lower body mass index and earlier onset of liver dysfunction. Studies used different criteria to define DILI and hepatic dysfunction, which may partly explain variability in outcomes. However, these findings are common features of patients with AAV in Japan and may increase susceptibility to DILI.^34^ Genetic predisposition may further amplify this risk, because the high prevalence of the *CYP3A5∗3* allele reduces CYP3A5 activity and increases reliance on CYP3A4 for avacopan metabolism, increasing hepatotoxicity risk with CYP3A4-modulating drugs.^35^ Inhibition of the C5aR may impair hepatic repair, because animal studies show aggravated injury and reduced hepatocyte regeneration in C5aR-deficient mice.^36^ Large-scale, pharmacovigilance studies are needed to confirm this potential pharmacogenetic susceptibility. Overall, it is important to perform cautious hepatic function monitoring, especially in this specific patient population. Dose adjustments may be necessary in older patients, alongside minimizing the use of concomitant medications.

This study has several limitations. First, it is a systematic review based on observational cohorts and case series; it mirrors real-world data and thus exhibits substantial heterogeneity across studies. Acknowledging the heterogeneity, particularly in safety signals and steroid withdrawal rates among the included studies, this reflects the diverse clinical scenarios encountered in the real-life management of AAV. Second, the absence of comparative studies and the unavailability of individual participant data precluded the conduct of an individual participant data meta-analysis, thereby limiting the statistical power of our findings. Third, the number of included studies and patients remains limited because of the recent introduction of avacopan and the limited access in many countries because of the costs of this treatment. Nonetheless, this study has notable strengths. It represents the first review analyzing real-world data on avacopan use in AAV treatment. By incorporating studies from diverse ethnic backgrounds, it reveals safety patterns not evident in randomized trials. Furthermore, despite the high rates of steroid withdrawal, the use of more flexible steroid dosing regimens may have contributed to more favorable treatment responses.

In conclusion, this analysis demonstrates the real-world effectiveness of avacopan in AAV treatment, with serious infection rates consistent with those observed in clinical trials. However, the high rate of hepatotoxicity observed in the Japanese studies warrants confirmation through larger, multiethnic, controlled studies.

## Disclosure

JIS’s work was supported by the Yonsei Fellowship, funded by Lee Youn Jae. AK reports grant or contracting fees from CSL Vifor and Otsuka; consulting fees from Amgen, Argenx, AstraZeneca, Boehringer-Ingelheim, CSL Vifor, Delta4, GSK, Miltenyi Biotec, Novartis, Novo Nordisk, Otsuka, Roche, Sobi, and Walden Biosciences; received support to attend meeting from AstraZeneca and Otsuka; and had a leadership role at IWG of European Renal Association. All the other authors declared no competing interests.

## Supplementary Material

Supplementary File (PDF) **Figure S1.** Forest plot showing steroid withdrawal rates of the included studies. **Table S1.** Quality assessment of included studies. **Table S2.** Ethnicity, ANCA status, and induction treatment characteristics from the included studies. **Table S3.** Generalized linear mixed models sensitivity analysis.

## Footnotes

Supplementary File (PDF)
**Figure S1.** Forest plot showing steroid withdrawal rates of the included studies. **Table S1.** Quality assessment of included studies. **Table S2.** Ethnicity, ANCA status, and induction treatment characteristics from the included studies. **Table S3.** Generalized linear mixed models sensitivity analysis.
