# Supplementary material for: Systematic Review of Efficacy and Safety of Avacopan in Real-World Clinical Practice
Source: Kidney Int Rep. 2025 Dec 31;11(4):103753. doi: 10.1016/j.ekir.2025.103753 (PMC12917383; doi:10.1016/j.ekir.2025.103753)

Supplementary table S1A. Summary of quality assessment of the included studies using NHLBI's tools for observational cohort and cross-sectional studies and case series studies.

Author	Rating
**Kubota**	Fair
**Falade**	Fair
**Draibe**	Fair
**Gabilan (2022)**	Good
**Uchida**	Fair
**Tagami**	Good
**Mori**	Fair
**Chalkia**	Good
**Gabilan (2024)**	Good
**Zonozi**	Good
**Zimmermann**	Good
**vanLeeuwen (2021)**	Good
**vanLeeuwen (2023)**	Good
**Eisinger**	Fair
**Takeuchi**	Good
**Assmann**	Fair

Supplementary table S1B. Quality assessment of case series studies.

**Case Series**	**Kubota**	**van Leeuwen (2021)**	**Chalkia**
Questions	**Rating**	**Rating**	**Rating**
**1. Was the study question or objective clearly stated?**	Yes	Yes	Yes
**2. Was the study population clearly and fully described, including a case definition?**	Yes	Yes	Yes
**3. Were the cases consecutive?**	Yes	Yes	Yes
**4. Were the subjects comparable?**	Yes	Yes	Yes
**5. Was the intervention clearly described?**	Yes	Yes	Yes
**6. Were the outcome measures clearly defined, valid, reliable, and implemented consistently across all study participants?**	Yes	Yes	Yes
**7. Was the length of follow-up adequate?**	No	Yes	No
**8. Were the statistical methods well-described?**	No	No	Yes
**9. Were the results well-described?**	CD	Yes	Yes
Overall	**Fair**	**Good**	**Good**

Abbreviation: CD: cannot determine.

Supplementary table S1C. Quality assessment of observational cohort and cross-sectional studies.

**Observational Cohorts**	**Falde**	**Draibe**	**Gabilan (2022)**	**Uchida**	**Tagami**	**Mori**	**Gabilan (2024)**	**Zonozi**	**Zimmermann**	**Van Leeuwen (2023)**	**Eisinger**	**Takeuchi**	**Assmann**
Questions	**Rating**	**Rating**	**Rating**	**Rating**	**Rating**	**Rating**	**Rating**	**Rating**	**Rating**	**Rating**	**Rating**	**Rating**	**Rating**
**1. Was the research question or objective in this paper clearly stated?**	Yes	Yes	Yes	Yes	Yes	Yes	Yes	Yes	Yes	Yes	Yes	Yes	Yes
**2. Was the study population clearly specified and defined?**	Yes	Yes	Yes	Yes	Yes	Yes	Yes	Yes	Yes	Yes	Yes	Yes	Yes
**3. Was the participation rate of eligible persons at least 50%?**	Yes	Yes	Yes	Yes	Yes	Yes	Yes	Yes	Yes	Yes	Yes	Yes	Yes
**4. Were all the subjects selected or recruited from the same or similar populations (including the same time period)? Were inclusion and exclusion criteria for being in the study prespecified and applied uniformly to all participants?**	Yes	Yes	Yes	Yes	Yes	Yes	Yes	Yes	Yes	Yes	Yes	Yes	Yes
**5. Was a sample size justification, power description, or variance and effect estimates provided?**	NA	Yes	NA	NA	NA	NA	NA	Yes	No	No	No	No	No
**6. For the analyses in this paper, were the exposure(s) of interest measured prior to the outcome(s) being measured?**	Yes	Yes	Yes	Yes	Yes	Yes	Yes	Yes	Yes	Yes	Yes	Yes	Yes
**7. Was the timeframe sufficient so that one could reasonably expect to see an association between exposure and outcome if it existed?**	No	Yes	Yes	No	Yes	No	Yes	Yes	Yes	Yes	Yes	Yes	Yes

**8. For exposures that can vary in amount or level, did the study examine different levels of the exposure as related to the outcome (e.g., categories of exposure, or exposure measured as continuous variable)?**	NA	NA	NA	NA	NA	NA	NA	NA	NA	NA	No	No	NA
**9. Were the exposure measures (independent variables) clearly defined, valid, reliable, and implemented consistently across all study participants?**	Yes	Yes	Yes	Yes	Yes	Yes	Yes	Yes	Yes	Yes	Yes	Yes	Yes
**10. Was the exposure(s) assessed more than once over time?**	NA	NA	NA	NA	NA	NA	NA	Yes	Yes	Yes	Yes	Yes	NA
**11. Were the outcome measures (dependent variables) clearly defined, valid, reliable, and implemented consistently across all study participants?**	Yes	Yes	Yes	Yes	Yes	Yes	Yes	Yes	Yes	Yes	Yes	Yes	Yes
**12. Were the outcome assessors blinded to the exposure status of participants?**	No	No	No	No	No	No	No	No	No	No	No	No	No
**13. Was loss to follow-up after baseline 20% or less?**	Yes	Yes	Yes	NA	Yes	NA	Yes	Yes	Yes	Yes	Yes	Yes	Yes
**14. Were key potential confounding variables measured and adjusted statistically for their impact on the relationship between exposure(s) and outcome(s)?**	No	No	No	No	No	No	No	No	No	No	NA	NA	NA
Overall	**Fair**	**Good**	**Good**	**Fair**	**Good**	**Fair**	**Good**	**Good**	**Good**	**Good**	**Good**	**Good**	**Fair**

Abbreviation: NA: not applicable.

Supplementary table S2. Ethnicity, ANCA status and induction treatment characteristics from the included studies.

Author	Japanese	White	Latino	Black African	MPO +	PR3 +	MPO+ & PR3+	ANCA + anti-GBM	ANCA negative	New diagnosis	PLEX	RTX (induction)	CYC (induction)	RTX + CYC (induction)	Pulse steroids
Tagami	21	0	0	0	18	3	0	0	0	18	NR	16	0	0	6
Mori et al	22	0	0	0	15	7	0	0	0	NR	NR	NR	NR	NR	NR
Draibe et al	0	24	3	2	21	8	0	0	0	17	7	14	2	12	17
Zimmermann et al	NR	NR	NR	NR	22	15	0	2	0	20	11	22	7	10	30
Uchida et al	36	0	0	0	30	3	0	0	0	20		23	1	2	13
Falde et al	NR	14	NR	NR	7	8	0	0	0	9	5	12	1	2	15
Gabilan et al	NR	NR	NR	NR	20	9	1	0	1	21	3	27	2	2	NR
Zonozi et al	NR	75	NR	NR	66	25	0	0	1	61	13	44	2	43	59
Chalkia et al	NR	NR	NR	NR	5	3	0	0	0	7	4	3	1	4	8
van Leeuwen et al	NR	NR	NR	NR	NR	NR	NR	NR	NR	NR	NR	NR	NR	NR	NR
Gabilan et al	NR	NR	NR	NR	7	1	1	0	0	5	0	8	0	0	1
van Leeuwen et al	NR	NR	NR	NR	4	4	0	0	0	0	0	1	0	0	0
Kubota et al	5	0	0	0	5	0	0	0	0	0	0	0	0	0	0
Takeuchi et al	57	0	0	0	45	11	NR	NR	NR	NR	NR	NR	NR	NR	NR

**Eisinger et al**	NR	NR	NR	NR	10	5	NR	NR	NR	10	5	12	1	2	NR
**Assmann et al**	NR	NR	NR	NR	10	20	NR	NR	NR	7	3	0	0	30	30

Abbreviations: ANCA: Anti-Neutrophil Cytoplasmic Antibody; MPO: Myeloperoxidase; PR3: Proteinase 3; Anti-GBM: Anti-Glomerular Basement Membrane; PLEX: Plasma Exchange; RTX: Rituximab; CYC: Cyclophosphamide; NR: Not reported.

Supplementary table S3. Generalized linear mixed models (GLMM) sensitivity analysis

**Outcome**	**k**	**tau2**	**pooled**	**lcl**	**ucl**
**Clinical remission at 6 months (n)**	9	3.769	0.924	0.724	0.982
**Clinical remission at 12 months (n)**	7	0	0.886	0.825	0.928
**Steroid withdrawal**	13	2.440	0.641	0.414	0.819
**Hepatotoxicity**	15	3.097	0.032	0.008	0.115
**Serious infections**	12	0	0.119	0.088	0.159

Supplementary figure S1. Forest plot showing steroid withdrawal rates of the included studies**.**

Study	Cluster	Events	Total	Proportion	95%-CI	Weight
Gabilan et al 2024	France	31	31	1.00	[0.89; 1.00]	3.5%
Gabilan et al 2022	France	4	9	0.44	[0.14; 0.79]	9.5%
Zimmermann et al 2024	Germany	15	39	0.38	[0.23; 0.55]	11.2%
Assman et al 2025	Germany	23	30	0.77	[0.58; 0.90]	9.9%
Tagami 2025	Japan	10	21	0.48	[0.26; 0.70]	9.6%
Mori et al 2024	Japan	.	22			0.0%
Uchida et al 2024	Japan	6	36	0.17	[0.06; 0.33]	9.5%
Kubota et al 2024	Japan	0	5	0.00	[0.00; 0.52]	2.5%
Takeuchi et al 2025	Japan	.	57			0.0%
van Leeuwen et al 2023	Mixed	.	30			0.0%
Draibe et al 2024	Other Europe	18	29	0.62	[0.42; 0.79]	10.1%
Chalkia et al 2024	Other Europe	6	8	0.75	[0.35; 0.97]	5.6%
van Leeuwen et al 2021	Other Europe	5	8	0.62	[0.24; 0.91]	6.3%
Falde et al 2024	US	10	15	0.67	[0.38; 0.88]	8.1%
Zonozi et al 2024	US	64	92	0.70	[0.59; 0.79]	11.8%
Eisinger et al 2025	US	15	15	1.00	[0.78; 1.00]	2.5%
**Random effects model**			**447**	**0.59**	**[0.42; 0.74]**	**100.0%**

Heterogeneity: $\tau^2 = 0.7615$, $p < 0.0001$

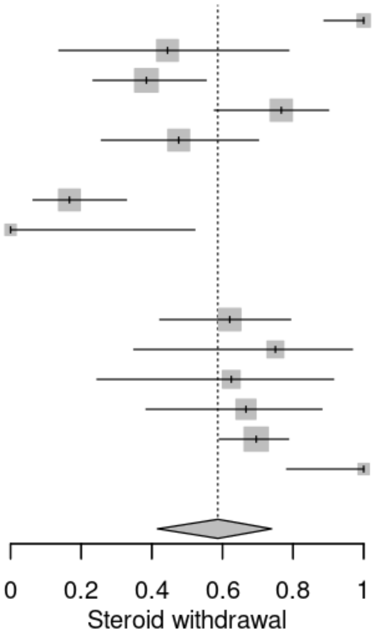

Supplement: Supplementary File (PDF) — Figure S1. Forest plot showing steroid withdrawal rates of the included studies. Table S1. Quality assessment of included studies. Table S2. Ethnicity, ANCA status, and induction treatment characteristics from the included studies. Table S3. Generalized linear mixed models sensitivity analysis. [file mmc1.pdf]
